# Role of PI3K-AKT-mTOR and Wnt Signaling Pathways in Transition of G1-S Phase of Cell Cycle in Cancer Cells

**DOI:** 10.3389/fonc.2013.00085

**Published:** 2013-04-12

**Authors:** Lakshmipathi Vadlakonda, Mukesh Pasupuleti, Reddanna Pallu

**Affiliations:** ^1^Department of Zoology, Kakatiya UniversityWarangal, Andhra Pradesh, India; ^2^SRM Research Institute, SRM UniversityKattankulathur, Tamil Nadu, India; ^3^National Institute of Animal Biotechnology, University of Hyderabad CampusHyderabad, India; ^4^Eicosanoids, Inflammation and Cancer Research Group, School of Life Sciences, University of HyderabadHyderabad, India

**Keywords:** mTORC1, autophagy, Wnt, G1-S, cell cycle

## Abstract

The PI3K-Akt pathway together with one of its downstream targets, the mechanistic target of rapamycin (mTOR; also known as the mammalian target of rapamycin) is a highly deregulated pathway in cancers. mTOR exists in two complexes, mTORC1 and mTORC2. Akt phosphorylated at T308 inhibits TSC1/2 complex to activate mTORC1; mTORC2 is recognized as the kinase phosphorylating Akt at S473. Inhibition of autophagy by mTORC1 was shown to rescue disheveled (Dvl) leading to activation of Wnt pathway. Cyclin D1 and the c-Myc are activated by the Wnt signaling. Cyclin D1 is a key player in initiation of cell cycle. c-Myc triggers metabolic reprograming in G1 phase of cell cycle, which also activates the transcription factors like FoxO and p53 that play key roles in promoting the progression of cell cycle. While the role of p53 in cancer cell metabolism in arresting glycolysis and inhibition of pentose phosphate pathway has come to be recognized, there are confusions in the literature on the role of FoxO and that of rictor. FoxO was shown to be the transcription factor of rictor, in addition to the cell cycle inhibitors like p21. Rictor has dual roles; inhibition of c-Myc and constitution of mTORC2, both of which are key factors in the exit of G1-S phase and entry into G2 phase of cell cycle. A model is presented in this article, which suggests that the PI3K-Akt-mTOR and Wnt pathways converge and regulate the progression of cell cycle through G0-G1-S-phases and reprogram the metabolism in cancer cells. This model is different from the conventional method of looking at individual pathways triggering the cell cycle.

## Introduction

The protein kinase B (*between* *protein* *kinase A and C*; Coffer and Woodgett, 1991), or Akt designated after the viral acute transforming retrovirus, Akt8 (Staal et al., 1977; Bellacosa et al., 1991; Downward, 1995) is recognized as the regulator of cell survival. Aberrant activation of the kinase is associated with many diseases, including cancer and diabetes (Pearce et al., 2010). Phosphorylation of Akt at threonine 308 (T308; in activation loop) and the serine 473 (S473; in hydrophobic motif) are considered important for its activity (Nicholson and Anderson, 2002). Akt is activated by insulin/insulin like growth factor (IGF) signaling (IIS). The autophosphorylation of the internal domains of IIS receptors leads to the recruitment of insulin receptor substrate (IRS) and activation of phosphatidylinositol 3-kinases (PI3K). PI3K phosphorylates phosphatidylinositol 4, 5 bisphosphate (PIP2) to PIP3 (Engelman et al., 2006; Manning and Cantley, 2007). PIP3 activates the phosphatidylinositol dependent protein kinase 1 (PDPK1) and recruits Akt to the plasma membrane. PDPK1 phosphorylates Akt T308 in the activation loop (Alessi et al., 1997). Several kinases, integrin-linked kinase (ILK), protein kinase Cα (PKCα), double-stranded DNA-dependent protein kinase (DNA-PK) ataxia telangiectasia mutated (ATM) gene product, and the mammalian target of rapamycin (mTOR) were proposed to phosphorylate Akt on Ser-473 (Dong and Liu, 2005). The mTORC2 (Sarbassov et al., 2005) is widely recognized as the key kinase that phosphorylates the Akt at S473. Ambiguity on the phosphorylation of this site however, remains; an atypical IκB kinase ε and TANK-binding kinase 1 (IKKε/TBK1) was reported to induce this phosphorylation in rictor (−/−) cells (Xie et al., 2011). There are reports that phosphorylation of Akt S473 could be cell specific (Riaz et al., 2012) or may not be required for full activation (Moore et al., 2011). T308 phosphorylation is considered a reliable biomarker of Akt activity especially for mTORC1 function (Jacinto et al., 2006; Breuleux et al., 2009; Vincent et al., 2011). Several tyrosine kinases are reported to phosphorylate Akt at different sites (Mahajan and Mahajan, 2012). Two phosphatases, the phosphatase and tensin homolog deleted from chromosome ten (PTEN) and the SH2 domain containing inositol-5-phosphatase 2 (SHIP2) regulate Akt function through dephosphorylation of 3-OH position of PIP3 (Leslie et al., 2003) and the 5-OH position (Aman et al., 1998) respectively.

*The mechanistic target of rapamycin* (mTOR: formerly known as mTOR; also known as FK506 binding protein 12-rapamycin associated protein 1 (FRAP1; Moore et al., 1996), in mammals exists in two multi protein complexes, mTORC1 and mTORC2, distinguished by their sensitivity to rapamycin. The catalytic cores of the two complexes have the kinase mTOR domain. While raptor (regulatory associated protein of mTOR) regulates the function of mTORC1, rictor (Rapamycin insensitive companion of mTOR) was shown to control the activity of mTORC2 (reviewed by Loewith et al., 2002; Laplante and Sabatini, 2009). DEPTOR is a negative regulator of the two complexes (Wang et al., 2012).

The complex mTORC1 responds to the nutrients and conditions that promote cellular growth. It is activated by AktT308 downstream of IIS (Wullschleger et al., 2006; Gamper and Powell, 2012). mTORC1 is activated both by the oncogenic PI3K-Akt as well as the Ras-Erk pathways, which inhibit the tuberous sclerosis complex (TSC1 and TSC2) (TSC complex) through the phosphorylation of the TSC2 (Manning and Cantley, 2007). The inhibition of TSC complex releases the inhibitory effect of TSC on the GTP-bound Rheb (Ras homolog enhanced in brain), which controls the activity of mTORC1. TSC is also inhibited by the Wnt pathway (Inoki et al., 2006). Activation of mTORC1 by amino acids is mediated by Rag GTPases. (Sancak et al., 2010), which is independent of IIS. AMP activated protein kinase (AMPK) inhibits mTORC1 by activating the TSC2 (Corradetti et al., 2004; Kwiatkowski and Manning, 2005; Inoki et al., 2006) and drugs that activate AMPK reverse the activation of mTORC1 (Guppy et al., 2011; He et al., 2011).

## mTORC1 is a Feedback Regulator of IIS Pathway and it also Regulates mTORC2

One of the key downstream targets of mTORC1, the p70 ribosomal S6 Kinase (S6K) phosphorylates IRS and inhibits the IIS in a feedback regulatory step (Zhang et al., 2008; Veilleux et al., 2010; Kang et al., 2011). An inverse relation is reported both in relative abundance and activation of mTORC1 and mTORC2 in cells (Sarbassov et al., 2004). S6K also phosphorylates rictor and inhibits mTORC2 assembly (Dibble et al., 2009; Julien et al., 2010; Treins et al., 2010).

S6K is also shown to inhibit glycogen synthase kinase3β (GSK3β) (Zhang et al., 2006). Recognized as one of the key targets of Akt, GSK3β was also shown to phosphorylate rictor (Chen et al., 2011). GSK3β has multiple roles ranging from glucose homeostasis (Kim and Kimmel, 2000) to inflammation (Wang et al., 2011), and it plays a key role in Wnt signaling (Wu and Pan, 2010). GSK3β phosphorylates the voltage-dependent anion channel (VDAC) and regulates the mitochondrial metabolite exchange and apoptosis (Shoshan-Barmatz et al., 2010); its depletion was shown to increase the beta cell proliferation (Stein et al., 2011). GSK3β cooperates with AMPK in activation of TSC complex that leads to inactivation of mTORC1 (Kwiatkowski and Manning, 2005).

Regulation of protein synthesis is recognized as one of the conserved role of mTORC1; it phosphorylates and inhibits, the eukaryotic initiation factor 4E (eIF4E)-binding proteins (4E-BP1/2), which are the inhibitors of translation (Castellvi et al., 2006; Ma and Blenis, 2009). The two functions of mTORC1, phosphorylation of S6kinase and inhibition of 4E-BP, have come to be accepted as routine markers for its activity and activation of protein synthesis in cells (Miron et al., 2003).

## RAS-Erk Map Kinase Signaling also Activates mTORC1

Over expression of epidermal growth factor receptors belonging to the proto-oncogene erbB (Thompson and Gill, 1985) and abberrant activation of RAS-Erk MAP kinase signaling was recognized as the cause of several cancers and antibodies targeting the receptors were developed during early 1980s (Sato et al., 1983; Schlessinger, 2000; Mendelsohn and Baselga, 2003; Lemmon and Schlessinger, 2010). The MAP kinase Erk was shown to phosphorylate and inactivate TSC2 (Ma et al., 2005) leading to activation of mTORC1. Several drugs that target the nutrient and growth factor (PI3K-Akt and Ras-Erk MAP kinase) pathways claim that the targeted drugs arrest the progression of cell cycle. The convergence of the two pathways at mTORC1 led to a surge in the activity in targeting of mTORC1 for the control of carcinogenesis. Rapamycin, which was initially recognized as an immunosuppressant for its ability to reduce organ rejection (Abraham and Wiederrecht, 1996) was subsequently found useful in treatment of cancers (Mita et al., 2003). But, the realization that rapamycin is inadequate in completely inhibiting mTORC1 functions (Shor et al., 2009) led to a search for the ATP competitive inhibitors (Bhagwat and Crew, 2010; Schenone et al., 2011). These inhibitors are claimed to arrest the cells in quiescent or gap1 (G0/G1) phase of the cell cycle (Evangelisti et al., 2011). But the exact link between the growth factor, mTOR pathways and cell cycle remains unexplained.

## Wnt Pathway is the Key Pathway in Activation of Cell Cycle

Wnt pathway is the key pathway in activation of cell cycle. Wnt signaling in general activates the Cyclin D, the *c-Myc*; matrix metalloproteinases, COX-2, peroxisome proliferator-activated receptors (PPARs), and the growth factors, and their receptors, and down regulates E-Cadherin, the cell cycle inhibitor P16ink4A (ARF) and p53 (http://www.stanford.edu/group/nusselab/cgi-bin/wnt/human_genetic_diseases; 2010). Wnt pathway thus, regulates the cancer cells entry into the cell cycle through the production of cyclin D. Cyclin D complexes with cyclin-dependent kinase 4/6 (Cdk4/6), inactivates the tumor suppressor protein retinoblastoma (Rb), and promotes the entry of the cell from G0 to G1 phase of cell cycle. E2F uncoupled from the phosphorylated Rb transcribes the cyclin E, which binds to Cdk2 and promotes the progression of the cell cycle. The up regulation of cyclin E/CDK2 is reported to correlate with the G1/S transition (Stott et al., 1998; Arima et al., 2004; Soto Martinez et al., 2005; Sun et al., 2007). Aberrant activation of Wnt pathway was shown to lead cells to malignant transformation (Polakis, 2012).

Activation of Wnt pathway is usually based on destabilizing the commonly known “destruction complex” comprising of the APC, the Axin, and the casein kinase I (CKI) and GSK3β. The disheveled (*Dvl*; Dsh gene homolog in mammals) protein is recognized as the key component in the signaling (Nusse, 2005; Gao and Chen, 2010).

## Autophagy Promotes Degradation of Dvl and Negatively Regulates the Wnt Pathway

The process of autophagy involves the fusion of phagophores with lysosomes (Yang and Klionsky, 2010); it plays a key role in human diseases like immune disorders (Deretic, 2011), neurodegenerative disorders (Weihl, 2011), and also in cancers (Brech et al., 2009; Stipanuk, 2009; White and Lowe, 2009; Chen and Klionsky, 2011; Nyfeler et al., 2011). mTORC1 was shown to inhibit autophagy by phosphorylation of ULK1 at Ser 757 (Kim et al., 2011).

Gao et al. (2010) demonstrated that autophagy is a negative regulator of the Wnt pathway by promoting the degradation of Dvl, a component of Wnt pathway. All the three isoforms of Dvl (Dvl1, Dvl2, and Dvl3) were shown to be degraded. Inhibition of autophagy by mTORC1 therefore, releases the Dvl. Autophagy mediated down regulation of Wnt signaling was confirmed by rapamycin treatment, which resulted in down regulation of the Wnt target genes *axin2*, *c-Myc*, and *cyclin D1*. Dvl appears to be the link for the cooperative interaction between the MAP kinase and PI3K-Akt-mTOR pathways converging at autophagy to activate cell proliferation. In addition, *c-Myc*, a downstream target of the Wnt signaling, was shown to be involved in carcinogenesis along with erbB2 as early as in 1980s (Dotto et al., 1986; Land et al., 1986). Pacheco-Pinedo et al. (2011) recently demonstrated that cooperation between K-Ras mutant and the Wnt/β-catenin signaling is the cause of aggressive lung tumor phenotype. Heallen et al. (2011) demonstrated that Hippo pathway inhibits the Wnt signaling to prevent cardiomyocyte proliferation; Dvl was shown to be the link between the two pathways. Apart from reusing Dvl from autophagic degradation, mTORC1 inactivation of GSK3β by S6K (Zhang et al., 2006) blocks the β-catenin degradation. Dvl was also shown to translocate into nucleus and in conjugation with the transcription factor of AP1 complex, c-jun is reported to stabilize the β-catenin-TCF/LEF signaling (Gan et al., 2008).

## Oncogenes and Tumor Suppressors are Both Involved in Metabolic Reprograming

**Figure 1:**
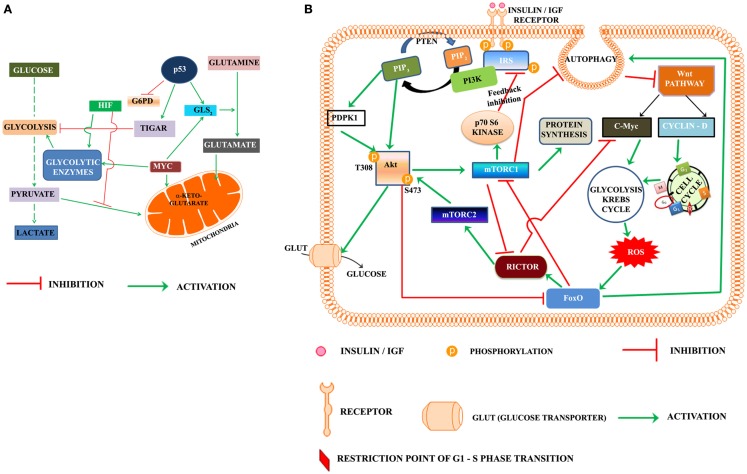
**(A)** Oncogenes and tumor suppressor modulate metabolic reprograming in cancer cells. HIF, hypoxia inducible factor; TIGAR, TP53-induced glycolysis and apoptosis regulator; MYC, Proto oncogene *c-Myc*; G6PD, glucose-6-phosphate dehydrogenase; GLS_2_, glutaminase 2. **(B)** Proposed model highlighting the role of PI3K-Akt-mTOR and Wnt pathways in regulation of the cell cycle progression in cancer cell: according to the presented model, activation of insulin/IGF receptor by nutrients/growth factors activates PI3K-Akt pathway. Akt phosphorylated on T308 activates mTORC1. One of the downstream target of mTORC1, the p70S6Kinase, phosphorylates the serine residues on IRS and inhibits the insulin/IGF signaling in a regulatory feedback control. The activated mTORC1 activates the protein synthesis, but inhibits autophagy. Inhibition of autophagy rescues Dvl and this lead to activation of Wnt pathway. The activated Wnt pathway up regulates cyclin D and *C-Myc*, which trigger the activation of cell cycle and metabolic reprograming. The metabolic activation in cancer cells leads to the production of reaction oxygen species (ROS), which activate the FoxO. FoxO is the transcription factor of rictor, one of the key components of mTORC2 recognized as the kinase phosphorylating Akt on S473. Rictor also inhibits c-Myc, which according to the present model is required for exit of G1/S restriction point. Akt, protein kinase B (T308, S473 are the phosphorylated sites Threonine 308 and Serine 473 of Akt), c-Myc is the oncoprotein activated by Wnt signaling, FoxO, fork head transcription factors of O group; GLUT, glucose transporter; IGF, insulin growth factor; IRS, insulin receptor substrate; mTORC1, 2, mammalian target of rapamycin (mTOR) Complex 1 and 2, PIP2, phosphoinositide 4,5 bisphosphate; PIP3, phosphoinositide 3,4,5 trisphosphate; rictor, a component of mTORC2; P70S6K, the p70 ribosomal S6K; PTEN, phosphatase and tensin homolog deleted from chromosome ten; ROS, reaction oxygen species; PDPK1 (also abbreviated as PDK1), phosphoinositide dependent protein kinase.

Progression of cell cycle also requires the activation of metabolic pathways in G1 phase. Glycolysis, Krebs cycle, and the pentose phosphate pathways are the key pathways involved in metabolic reprograming of cells. Warburg, in the early twentieth century, was the first to suggest that cancer cells utilize the aerobic glycolysis for promotion of tumorigenesis (Warburg, 1956). A re-examination of Warburg hypothesis in the last part of twentieth century led to a search for the role of oncogenes and tumor suppressors in metabolism (Figure 1A). Akt was named as the “Warburg enzyme” (Robey and Hay, 2009), p53 was recognized to suppress both glycolysis through TIGAR (*TP53*-induced glycolysis and apoptosis regulator; Bensaad et al., 2006) and Pentose phosphate pathway by inhibiting the G6PD (Jiang et al., 2011). It was shown to activate glutamine metabolism and control the ROS production (Gottlieb, 2011; Maddocks and Vousden, 2011). HIF and *c-Myc* were shown to up regulate the enzymes of glycolysis (Kim et al., 2007). The signature of cancer cells is recognized by loss of function of the tumor suppressors p53, PTEN and by activation of Akt, Myc, HIF-1α, and NFkB (Markert et al., 2012). The oncogene *c-Myc* is recognized to play an important role in activation of genes of enzymes of glycolysis and Krebs cycle as well as those involved in chromatin structure, and its transcriptional networks that are involved predominantly in cell cycle regulation and cellular metabolism and protein synthesis specific to the G0-G1-S transition in cancer cells, lymphocytes, and in embryonic stem cell (Kim et al., 2010; Swami, 2010; Lin et al., 2012).

## The Role of FoxO Transcription Factors

One of the consequences of metabolic reprograming in cancer cells is activation of FoxO transcription factors (Ronnebaum and Patterson, 2012). The FoxO transcription factors are reported to sequester β-catenin away from the TCF/LEF transcription factors (Hoogeboom et al., 2008; Hoogeboom and Burgering, 2009). Although the exact mechanism is still enigmatic, deregulation of adherens junction (AJ) was reported to result in translocation of the FoxO transcription factors into nucleus (Fournier et al., 2009). One of the critical steps in the progression of cell cycle is the crossover of restriction point in G1, which is regulated by the E2F-pRb. The role of FoxO in up regulating cell cycle inhibitors p15 (INK4b) and p19 (INK4d) is viewed as an arrest of cell cycle (Katayama et al., 2008). Rictor transcribed by FoxO is a key component of the mTORC2 (Chen et al., 2010). Guo et al. (2012) reported that rictor promotes ubiquitination and degradation of c-Myc and cyclin E and suggested that it leads to the arrest of cell cycle in G1 phase. Rictor is also reported to be involved in regulation of Rho GTPases (Jacinto et al., 2004) and RhoA activation is crucial for G1-S progression of cell cycle (Zhang et al., 2009). The up regulation of cell cycle inhibitors (p21WAF1/CIP1 and p27KIP1) and transcription of rictor by FoxOs and inhibition of c-Myc and cyclin E reported by Guo et al. (2012) should therefore, be viewed as a regulation of the exit of G1/S phase of cell cycle rather than inhibition of cell cycle in G1 phase. FoxO is also reported to transcribe the antioxidant genes like sestrins (Chen et al., 2010; Lee et al., 2010) which inhibit mTORC1 and inhibit the mitochondrial metabolism (Ferber et al., 2012). Young et al. (2009) demonstrated a feedback link between mTORC1 and C2 signaling and the timing of inhibition of mTORC1 correlated with activation of autophagy and cyclin A. These results suggest the role of FoxO in progression of cell cycle and assembly of mTORC2. mTORC2 was shown to be required for proliferation and survival of TSC2-Null cells (Goncharova et al., 2011). The hypothesis that FoxOs are involved in the progression of cell cycle is further strengthened by the fact that FoxOs also regulate the expression of mitotic genes such as cyclin B, polo-like kinase (Plk) (Alvarez et al., 2001). In addition, recent reports indicate the role of FoxO1 in dedifferentiation of pancreatic β-cells (Talchai et al., 2012) and in osteoblast proliferation (Kode et al., 2012).

## The Proposed Model

Based on the foregone discussion, we propose that activation of PI3K-Akt-mTORC1 leads to inhibition of autophagy and rescues Dvl, which activates the Wnt pathway (Figure 1B). The transcriptional activation of *Cyclin D* by Wnt pathway triggers the entry of cells from G0 to G1 phase. *c-Myc* promotes reprograming of cancer cell metabolism in the G1 phase, which apart from generating ROS, activates the transcription factors like p53 and FoxO and autophagy. The transcription of rictor by FoxO leads to the inhibition of *c-Myc* and promotes exit of the restriction point of G1-S phase of cell cycle. Rictor also constitutes mTORC2 in G2 phase. Phosphorylation of Akt at S473 by mTORC2 leads to feedback inhibition of FoxO.

## Some Unanswered Questions

Cancer cells consume lots of glucose, but it is reported that glucose transporters are activated only following phosphorylation of Akt at S473 (Kumar et al., 2010) and it coincides with the inactivation of FoxO proteins. Under hypoxic conditions, loss of p53 promotes the expression of mono carboxylate transporters (MCT1) and lactate export which is reported to promote cell proliferation by fueling mitochondrial respiration (Boidot et al., 2012). Do cancer cells exiting the divisional phase depend on excess glucose and glycolytic flux fuels respiration, while actively proliferating cells depend on lactate as a fuel resulting in a Warburg effect?

## Conflict of Interest Statement

The authors declare that the research was conducted in the absence of any commercial or financial relationships that could be construed as a potential conflict of interest.

## Abbreviations

Akt, protein kinase B (T308, S473 – phosphorylated sites Threonine 308 and Serine 473); APC, adenomatous polyposis coli; AXIN, axis inhibition protein; CDK2, cyclin-dependent kinase 2; CK, casein kinase; CKI, cyclin-dependent kinase inhibitors; c-Myc, the oncoprotein activated by Wnt signaling; COX-2, cyclooxygenase-2; Dvl, Dsh homolog in mammals; 4E-BP, eukaryotic translation initiation factor (eIF4E) binding protein1; FoxO, fork head transcription factors of O group; G0, G1, and S are phases of cell cycle; GLUT, glucose transporter; GSK3β, glycogen synthase kinase3β; HIF, hypoxia inducible factor; IGF, insulin like growth factor; IIS, insulin/insulin like growth factor signaling; IRS, insulin receptor substrate; LEF, lymphoid enhancer-binding factor; LRP, LDL receptor protein; mTORC1, 2, mechanistic target of rapamycin complex 1 and 2 (mTOR, formerly known as mammalian target of rapamycin); p16INK 4a ARF, cyclin-dependent kinase inhibitor 2A family of cell cycle inhibitors; p16INK 4a^ARF^, cyclin-dependent kinase inhibitor 2A family of cell cycle inhibitors; PDPK1, phosphoinositide dependent kinase 1 (the abbreviation PDPK1 is preferred over the original PDK1 in the article to avoid confusion with the pyruvate dehydrogenase kinase, which is also abbreviated as PDK1 in the literature); PDK2, phosphoinositide dependent kinase2 (a putative kinase thought to phosphorylate Akt on S473); PI3K, phosphatidylinositol 3-kinases; PIP2, phosphatidylinositol 4,5 bisphosphate; PIP3, phosphatidylinositol 3,4,5 trisphosphate; PPP, pentose phosphate pathway; *PTEN*, phosphatase and tensin homolog deleted from chromosome ten; rictor, a component of mTORC2; ROS, reaction oxygen species; S6K, the p70 ribosomal S6K; sestrins, stress response proteins; snail/slug, transcriptional inhibitors of E-Cadherin; TCF, T-cell factor; TIGAR, *TP53*-induced glycolysis and apoptosis regulator.
